# Propofol Suppresses Cell Progression by Inhibiting CCL18 Expression in Hepatoblastoma

**DOI:** 10.1155/2021/6880473

**Published:** 2021-07-26

**Authors:** Hua Zhang, Pingling Lin, Lei Fu, Zhijun Li, Yan Ding

**Affiliations:** ^1^Department of Pharmacy, Yantaishan Hospital, Yantai 264000, China; ^2^Department of Pediatrics (I), Jiyang People's Hospital, Jinan 251400, China; ^3^PIVAS, Qingdao Central Hospital Affiliated to Qingdao University, Qingdao 266042, China; ^4^Department of Pediatrics, Zhangqiu District People's Hospital, Jinan 250200, China; ^5^Department of Pediatric Ward (II), Rizhao People's Hospital, Rizhao 276800, China

## Abstract

**Background:**

Propofol is an anesthetic commonly used clinically and has been found to have antitumor activity in various cancers. The purpose of this study was to investigate the role of propofol in hepatoblastoma (HB).

**Methods:**

CCK-8 and transwell were used to measure cell proliferation, migration, and invasion in HB cells. Cell apoptosis rate was measured by FCM. The expression of CCL18 in HB tissues and cells was detected by RT-qPCR. Western blotting was used to explore the protein expression of CCK18- and PI3K/AKT-related proteins.

**Results:**

The expression of CCL18 in HB tissues and cells was overexpressed compared with control groups. CCL18 knockdown was found to notably block cell proliferation and progression, while enhancing cell apoptosis in HuH-6 and HepT1 cells. Furthermore, propofol suppressed the proliferation of HB cells in a dose-dependent manner. According to the results, we chose 5 *μ*g/mL of propofol-treated cells for 48 hours as the subsequent experimental conditions. We found that propofol (5 *μ*g/mL, 48 h) significantly blocked cell migration and invasion, but induced cell apoptosis in HuH-6 and HepT1 cells. In addition, CCK18 overexpression facilitated cell progression in HB cells, while propofol dramatically suppressed the effect of CCK18. Besides that, propofol suppressed the PI3K/AKT pathway.

**Conclusion:**

Propofol suppressed the development of HB cells by inhibiting CCK18 expression and the PI3K/AKT pathway. Therefore, we infer that propofol plays a role in the treatment of HB.

## 1. Introduction

Hepatoblastoma (HB) is mainly derived from human immature liver precursor cells [1]. HB is the most common primary liver malignant tumor in children, and 90% of patients are affected before the age of 5 years [2]. HB is an increasingly common tumor worldwide, especially in North America and Europe. HB is treated by adjuvant chemotherapy, radiotherapy, and transplantation, and the survival rate for patients with HB has increased dramatically. However, for patients with advanced HB, the prognosis is still poor [3]. Therefore, it is essential to explore the early biomarkers of HB and find effective treatment methods for patients with HB.

Propofol is a short-acting intravenous anesthetic of alkyl phenols, which is widely used in clinical practice [4]. It plays a sedative and hypnotic role by activating the GABA receptor-chloride ion complex [5]. Propofol has the advantages of rapid onset of anesthesia induction, rapid recovery and complete functional recovery, and low incidence of postoperative malignant vomiting [6]. In addition to the anesthetic effect, propofol can also affect the biological process of tumors and have different effects on different tumor cells [7]. Propofol has been reported to affect the epigenetic pathways such as lncRNAs, miRNAs, and cancer-related proteins and regulate the genetic signaling pathways such as MAPK, PI3K/AKT, NF-*κ*B, SLUG, and Nrf2 [8]. A growing number of studies have shown that propofol may exert an antitumor effect in human tumors, such as colon cancer [9], ovarian cancer [10], and lung cancer [11]. At present, propofol is widely used in HB surgery and postoperative sedation. Therefore, the effect of propofol on the biological behavior of HB cells has gradually attracted researchers' attention.

CCL18 is a member of the chemokines family. CCL18 has been found to play a special role in the development of human tumors. It is reported that the role of CCL18 in tumors appears after TAM involvement into the tumor niche [12]. Lane et al. reported that CCL18 in ascites accelerated cell migration by regulating Pyk2 in ovarian cancer [13]. In oral cancer, CCL18 was found to facilitate cell progression by activating the JAK2/STAT3 pathway [14]. However, the role of CCL18 in HB is not well understood.

In this study, we aimed to investigate the effect of propofol on the progression of HB. Furthermore, propofol was confirmed to suppress HB tumor progression by regulating CCL18.

## 2. Materials and Methods

### 2.1. Patient Samples

HB tissues were obtained from 20 children in Yantaishan Hospital (Yantai, China). All patients were diagnosed as HB preoperatively. Twenty paracancer tissues were at least 2 cm from the tumor margin. All specimens were placed in liquid nitrogen within 30 minutes after isolation and then stored in a −80°C refrigerator. This study was approved by the ethics committee of Yantaishan Hospital, and informed consent of the patients was obtained before surgery.

### 2.2. Cell Lines and Propofol

Human HB cell lines HepG2, HuH-6, and HepT1 and normal liver cells MIHA were collected from Yantaishan Hospital. After resuscitation at 37°C, the cells were resuspended on Dulbecco's Modified Eagle Medium (DMEM) containing 100 ml/L fetal bovine serum (FBS) and 10 ml/L penicillin and streptomycin. The cells were cultured in a constant-temperature incubator at 37°C and 5% CO_2_ for 3-4 days. After cell fusion, the cells were subcultured into a cell culture flask (5 ml/L). Cells were cultured for 4-5 generations for subsequent experiments. Propofol was obtained from Qingyuan JiaBo Pharmaceutical Co. Ltd. (China). The final concentrations of propofol were 0, 2, 5, and 10 *μ*g/mL with complete medium.

### 2.3. Transfection of si-CCL18

Cells (1 × 10^6^/mL) were inoculated into a 6-well plate. HuH-6 and HepT1 cells were transfected with CCL18 si-RNA (5′-ACAAGTTGGTACCAACAAATT-3′), pcDNA3.1-CCL18, and the corresponding negative control oligonucleotides mixed with LipofectamineTM 2000, respectively. After transfection for 48 h, the cells were collected for subsequent experiments.

### 2.4. Cell Proliferation

Proliferative ability of HuH-6 and HepT1 cells was evaluated by using the cell counting kit-8 (CCK-8) method. Cell suspension was evenly planted in a 96-well plate with a density of 3х 10^3^ cells/well. Cells were cultured in an incubator for 24, 48, and 72 h and then added with 100 *μ*l DMEM medium and 10 *μ*l CCK-8 solution. The cells were replaced in the incubator for further incubation for 1 to 4 hours. A microplate analyzer was used to detect the OD value of cells at 450 nm.

### 2.5. Cell Migration and Invasion

Transwell assay was performed to measure cell migration and invasion in HuH-6 and HepT1 cells. Cells are routinely digested to prepare cell suspensions. 100 *μ*l cell suspension was inoculated in the upper transwell chamber, and 500 *μ*l DMEM was added to the lower chamber. The cells were cultured in a constant-temperature cell incubator a 37°C and 5% CO_2_. After 24 hours, 200 *μ*l paraformaldehyde was added to fix cells for 15 min. Then, cells were stained with crystal violet for 20 min. Photographs were taken under a microscope, and the number of migrated and invaded cells was counted.

### 2.6. Cell Apoptosis

Flow cytometry (FCM) was used to measure cell apoptosis in HuH-6 and HepT1 cells. Cells were seeded into a 24-well plate and incubated in an incubator at 37°C and 5% CO_2_ for 24 h. After centrifugation at 1500 r/min for 5 min, the supernatant was discarded. Cells were labeled with 1 mL Annexin V and 1 mL Sytox Green. After incubation at 37°C for 15 min, FCM (Beckman Coulter, Germany) was performed to detect cell apoptosis rate within 4 h.

### 2.7. Real-Time PCR

Total RNA was extracted by Trizol reagent. Reverse transcription system: 5xPrimer Script Buffer 2 *μ*l, RT Enzyme Mix 0.5 *μ*l, primer 0.5 *μ*l, RNA1 *μ*l, and RNAase-free ddH_2_O 6 *μ*l. The cDNA was obtained by reverse transcription and then diluted to 50 ng/*μ*l. Real-time PCR transcription system: SYBR premix ExTaqTM II (2x) 10 *μ*l, forward primer 1 *μ*l, reverse primer 1 *μ*l, cDNA 1 *μ*l, and ddH2O 7 *μ*l. The primers were CCL18 forward: 5′-TGGCAGATTCCACAAAAGTTCA-3′, reverse 5′-GGATGACACCTGGCTTGGG-3′; GAPDH forward 5′-GCACCGTCAAGGCTGAGAAC-3′, and reverse 5′-TGGTGAAGACGCCAGTGGA-3′.

### 2.8. Western Blotting

After transfection for 24–48 hours, cell proteins were extracted by RIPA lysate. The BCA kit was used to detect protein concentration and purity. The protein samples were subjected to SDS-PAGE gel electrophoresis at 80 V and for 2 hours. After the protein was transferred to the PVDF membrane, it was sealed with milk powder at room temperature for 1–2 hours. Subsequently, the PVDF membrane was added with primary antibody and incubated overnight at 4°C. Then, the PVDF membrane was incubated with secondary antibody at room temperature for 2 hours. Finally, ECL chemiluminescence solution was added into the gel imaging system to collect images, and the protein bands were quantitatively analyzed with Image J.

### 2.9. Statistical Analysis

SPSS23.0 statistical software and GraphPadPrism software were used for statistical analysis. The *t*-test was used to compare the differences between the two experimental groups. Univariate ANOVA analysis was used for comparison between groups. *p* < 0.05 indicated that the comparison results between the experimental groups were statistically significant. All data are mean ± SD from three or more independent trials.

## 3. Results

### 3.1. Overexpression of CCL18 Was Observed in HB

**Figure 1:**
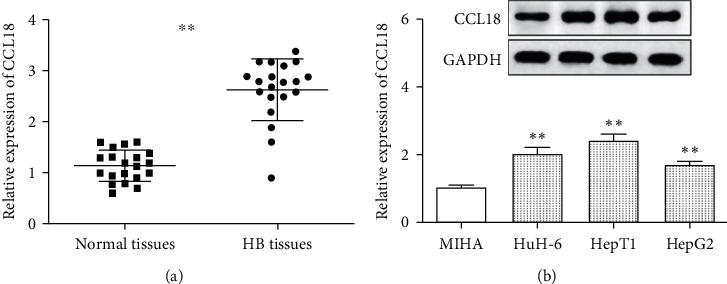
Overexpression of CCL18 was observed in HB. (a) CCL18 was significantly upregulated in 20 HB tissues compared with normal tissues. (b) CCL18 was overexpressed in HuH-6, HepT1, and HepG2 cells compared with MIHA cells. ^∗∗^*p* < 0.01.

According to the RT-qPCR assay, tumor tissues assumed the overexpression of CCL18 compared with normal tissues (Figure 1(a)). Next, western blot results showed that CCL18 was higher expressed in HepG2, HuH-6, and HepT1 cells than in MIHA cells (Figure 1(b)). Therefore, we speculated that CCL18 might play a specific role in HB progression.

### 3.2. CCL18 Depletion Played an Antitumor Role in HuH-6 and HepT1 Cells

**Figure 2:**
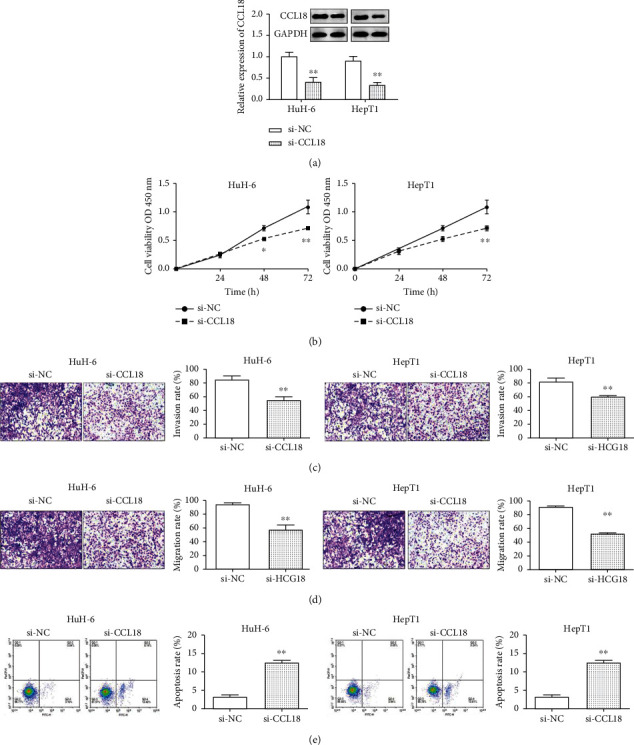
CCL18 depletion played an antitumor role in HuH-6 and HepT1 cells. (a) The expression of CCL18 in HuH-6 and HepT1 cells transfected with CCL18 si-RNA. (b), Cell proliferation was notably suppressed by CCL18 knockdown in HuH-6 and HepT1 cells. (c) Cell invasion was notably inhibited by CCL18 knockdown in HuH-6 and HepT1 cells (scale bar = 100 *μ*m). (d) Cell migration was notably suppressed by CCL18 knockdown in HuH-6 and HepT1 cells (scale bar = 100 *μ*m). (e) Cell apoptosis was notably promoted by CCL18 knockdown in HuH-6 and HepT1 cells. ^∗∗^*p* < 0.01.

To investigate the specific role of CCL18 in HB, we knock down its expression in HuH-6 and HepT1 cells. As shown in Figure 2(a), the expression of CCL18 was notably reduced by CCL18 si-RNA. CCK-8, transwell, and FCM were used to assess the effect of CCL18 knockdown in HB cells. As we expected, cell proliferation was significantly declined in HuH-6 and HepT1 cells when transfected with CCL18 knockdown (Figure 2(b)). Furthermore, after CCL18 knockdown transfection, the invasiveness of HuH-6 and HepT1 cells was significantly decreased (Figure 2(c)). Similarly, the migration ability of HuH-6 and HepT1 cells transfected with CCL18 knockdown was notably reduced (Figure 2(d)), whereas FCM results verified that CCL18 depletion significantly accelerated cell apoptosis in HB cells (Figure 2(e)). Taken together, we assumed that CCL18 knockdown played a role as a tumor suppressor in HB cells.

### 3.3. Propofol Suppressed Cell Growth, Migration, and Invasion in HB Cells

**Figure 3:**
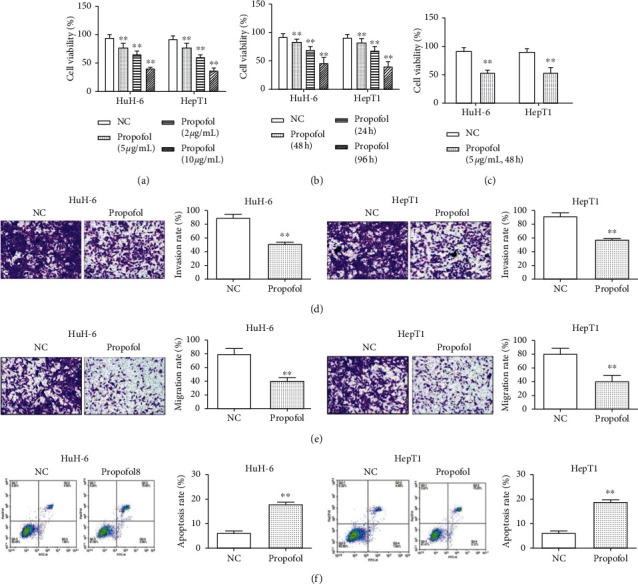
Propofol suppressed cell growth, migration, and invasion in HB cells. (a) The effect of different concentrations of propofol (0, 2, 5, and 10 *μ*g/mL) on HB cell viability. (b) The effect of different treatment time of propofol (0, 24, 48, and 96 h) on HB cell viability. (c) Propofol (5 *μ*g/mL, 48 h) suppressed cell viability in HuH-6 and HepT1 cells. (d) Propofol (5 *μ*g/mL, 48 h) suppressed cell invasion in HuH-6 and HepT1 cells (scale bar = 100 *μ*m). (e) Propofol (5 *μ*g/mL, 48 h) inhibited cell migration in HuH-6 and HepT1 cells (scale bar = 100 *μ*m). (f) Propofol (5 *μ*g/mL, 48 h) enhances cell apoptosis in HuH-6 and HepT1 cells. ^∗∗^*p* < 0.01.

To investigate the effect of propofol in HB cells, the concentration and treatment time of propofol were first determined by CCK-8 results. We found that cell viability of HuH-6 and HepT1 cells was notably reduced in a concentration-dependent effect of propofol (0, 2, 5, and 10 *μ*g/mL) (Figure 3(a)). Next, cell viability was suppressed in HuH-6 and HepT1 cells treated with 5 *μ*g/mL propofol for 24, 48, or 96 h (Figure 3(b)). According to the experimental results, 5 *μ*g/mL propofol treatment for 48 hours (*p* < 0.01) was selected for the subsequent experimental condition. CCK-8 assay indicated that propofol (5 *μ*g/mL, 48 h) suppressed cell viability in HB cells (Figure 3(c)). Transwell assay displayed that propofol (5 *μ*g/mL, 48 h) significantly reduced cell invasion (Figure 3(d)). Not surprisingly, cell migration ability was notably reduced by propofol (Figure 3(e)). In addition, propofol dramatically accelerated cell apoptosis in HuH-6 and HepT1 cells (Figure 3(f)).

### 3.4. Propofol Suppressed Cell Progression by Inhibiting CCL18 Expression and Downregulating the PI3K/AKT Pathway in HB

**Figure 4:**
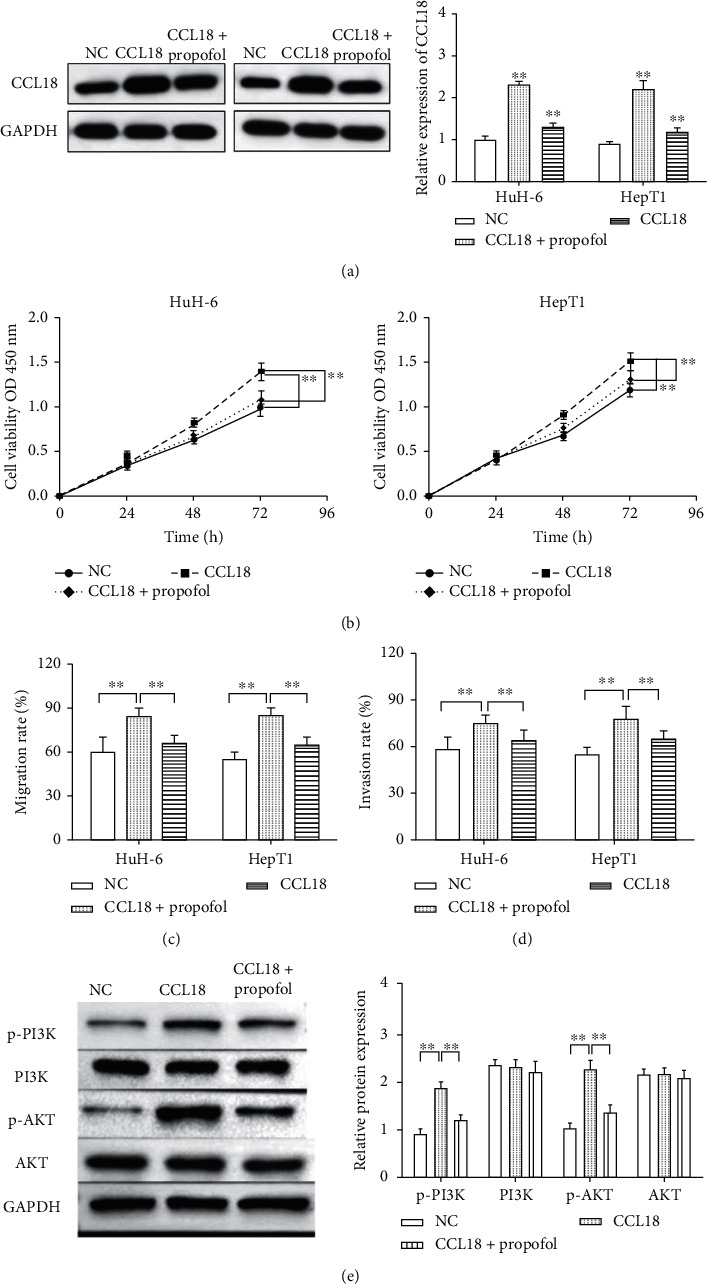
Propofol suppressed cell progression by inhibiting CCL18 expression and downregulating the PI3K/AKT pathway in HB. (a) The expression of CCL18 after treated with propofol. (b) After treated with propofol, cell proliferation induced by CCL18 was suppressed. (c)-(d) After propofol, cell invasion and migration promoted by CCL18 was decreased. (e) CCL18 vector promoted the phosphorylation of PI3K and AKT, while propofol suppressed the phosphorylation of PI3K and AKT. ^∗∗^*p* < 0.01.

In order to study the effect of propofol on CCL18 in the progression of HB cells, HuH-6 and HepT1 cells transfected with CCL18 vector were treated with propofol. As shown in Figure 4(a), propofol decreased the expression of CCL18 vector in HuH-6 and HepT1 cells. Next, functional experiments were performed to verify the interaction between propofol and CCL18 in HB cells. We noticed that CCL18 vector accelerated the proliferation of HB cells, while propofol weakened the effect of CCL18 (Figure 4(b)). Likewise, propofol neutralized the stimulative effect of CCL18 on cell migration and invasion (Figures 4(c) and 4(d)).

The PI3K-Akt pathway is a classic antiapoptotic and prosurvival signal transduction pathway, which plays an important role in the occurrence, proliferation, invasion, and metastasis of tumors [15]. Western blot assay was used to study the effect of propofol/CCL18 on the PI3K/AKT pathway. The results displayed that CCL18 vector increased the phosphorylation of PI3K and AKT, while propofol suppressed the phosphorylation of PI3K and AKT (Figure 4(e)).

## 4. Discussion

The expression and function of CCL18 in HB were evaluated in this study. We found that CCL18 was obviously overexpressed in HB tissues. Additionally, CCL18 was also overexpressed in HB cell lines (HepG2, HuH-6, and HepT1 cells) compared with MIHA cells. CCK-8, transwell, and FCM assay were used to study the effect of CCL18 on tumor progression in vitro. In HuH-6 and HepT1 cells, knockdown of CCL18 significantly reduced cell proliferation, invasion, and migration, but significantly promoted cell apoptosis. Combined with the experimental results, we speculated that CCL18 plays a role as a procancer factor in HB. Many previous studies have confirmed that CCL18 plays an oncogenic role in a variety of cancers. Lin et al. found that CCL18 facilitated cell progression and EMT by regulating the NF-*κ*B pathway in hepatocellular carcinoma [16], which is consistent with our findings. Furthermore, CCL18 promoted cell growth and cell motility in squamous cell carcinoma of the head and neck [17]. In osteosarcoma, Su et al. confirmed that CCL18 promoted tumor progression by increasing UCA1 expression [18].

Propofol is the most widely used intravenous anesthetic in clinical anesthesia. Propofol is characterized by quick action, strong effect, and quick recovery. At present, the role of propofol in human tumors has become a research hotspot. Previous studies have shown that propofol obviously suppresses tumor development. Zhou et al. reported that propofol inhibited tumor growth in mice with liver cancer [19]. Propofol was verified to reduce proinflammatory factors and increase anti-inflammatory factors, so as to effectively improve the immune function of patients with pancreatic cancer [20]. In pancreatic cancer, propofol suppressed cell proliferation and migration by inhibiting the ERK/MMPs pathway [21]. In hepatocellular carcinoma, propofol has been shown to block the progression [22–24]. Similarly, we found that propofol inhibited extracellular activity in HB. Unlike the previous results, we studied the effect of propofol on CCL18 expression in HB cells. We noticed that propofol reduced the expression level of CCL18. Above all, the promotional effect of CCL18 in HB was suppressed by propofol.

The PI3K/AKT pathway is a classical signaling pathway involved in the regulation of various cellular functions such as proliferation, differentiation, apoptosis, and glucose transport [25]. In recent years, the PI3K/AKT signaling pathway has been found to be closely related to the occurrence and development of human tumors [26, 27]. This pathway regulates the proliferation and survival of tumor cells and is related to tumor cell migration, adhesion, angiogenesis, and degradation of the extracellular matrix [28, 29]. Furthermore, propofol was found to suppress the phosphorylation of PI3K and AKT. Therefore, the results showed that propofol/CCK18 suppressed the development by inhibiting the PI3K/AKT pathway.

To sum up, we found that propofol notably suppressed the progression in HB cells. In addition, propofol played an inhibitory role by blocking the CCL18 expression and PI3K/AKT pathway in HB. However, the dual effect of propofol in relieving the pain of tumor patients and inhibiting tumor progression needs to be confirmed by animal models and clinical trials.

## Data Availability

The data used to support the findings of this study are available from the corresponding author upon request.
